# Late onset epilepsia partialis continua in a middle-aged patient with huge arachnoid cyst

**DOI:** 10.22088/cjim.12.0.464

**Published:** 2021

**Authors:** Mohammad Javad Nasr, Amir Hossein Zohrevand, Ali Alizadeh khatir

**Affiliations:** 1 Student Research Committee, Babol University of Medical Sciences, Babol, Iran; 2 Department of Neurosurgery, School of Medicine, Babol University of Medical Sciences, Babol, Iran; 3 Department of Neurology, School of Medicine, Babol University of Medical Sciences, Babol, Iran; 4 Mobility Impairment Research Center, Health Research Institute, Babol University of Medical Sciences, Babol, Iran; 5 5.Clinical Research Development Unit of Ayatollah Rouhani Hospital, Babol University of Medical Sciences, Babol, Iran

**Keywords:** Arachnoid cyst, Epilepsia partialis continua, Spastic cerebral palsy

## Abstract

**Background:**

Arachnoid cysts are congenital or acquired cerebrospinal fluid (CSF) filled intra arachnoidal lesions, included 1% of all infantile intracranial masses and were discovered incidentally in MRI or CT-scan. The vast majority of these lesions are generally asymptomatic but some patients with arachnoid cyst have headache, dizziness, seizure (or epilepsy), vestibular symptoms and cognitive impairment.

**Case Presentation:**

We present a case of a 43-year-old woman who has late onset epilepsia partialis continua and had right spastic cerebral palsy due to huge arachnoid cyst. Surprisingly without any history of seizure, her first seizure presents with sustained seizures (epilepsia partialis continua) and occur in the middle age for the first time.

**Conclusion:**

Most arachnoid cysts are asymptomatic and may not produce any symptoms throughout life. In our case, the late onset epilepsia partialis continua in the 5^th^ decade of life with probably a large arachnoid cyst without any history of seizure before that is unusual. Conservative approaches usually made for the management of arachnoid cysts as patients with these cysts usually maintain the vital neurological functions.

In 1831, Bright described what an arachnoid cyst is (1). Arachnoid cyst is an intra-arachnoidal cerebrospinal fluid compression, which is covered by arachnoidal cells and collagen (2). This rare central nervous system malformation included approximately 1% of all atraumatic intracranial mass lesions (3). It has congenital or acquired origin (such as tumor, trauma or infection) and is discoverable at an early age (2-40. Most arachnoid cysts are asymptomatic and they are discovered incidentally in MRI images and usually at an early age (5-7). Headache and seizure have been seen in patients with arachnoid cyst (3). Although patients complain of seizure (however it is less than headache and vestibular symptoms), but there is no clear relation between arachnoid cyst and these symptoms (and other like dizziness or cognitive impairment) (8, 9). Asymptomatic cysts are managed conservatively and the patient with symptomatic cyst gets surgery or other ways of treatment (10, 11). In this case, we aimed to present late onset epilepsia partialis continua in a case with right spastic cerebral palsy due to large arachnoid cyst. 

## Case presentation

A 43-year-old woman with spastic cerebral palsy presented to the emergency department having sustained multiple complex partial seizures (epilepsia partialis continua) since morning.

Her family denied any previous seizure nor traumatic head injury before then. She had right spastic cerebral palsy with right hemiparesia from infantile but no infantile hypoxia and neonatal infection. The family history for epilepsy was negative. There was not any chronic disease and she had negative drug history. The neurological examination at the time of admission, revealed right spastic hemiparesia with motor power grade 2/5. Also, thedeep tendon reflexes in the right limbs exaggerated and the right Babinski sign was detected. Her blood biochemistry analysis was unremarkable. An urgent non-contrast brain CT-scan revealed a highly abounded hypodense area in the left hemisphere. After that, she underwent brain magnetic resonance imaging that revealed an arachnoid cyst, with compressive effect on the left cerebral hemisphere. It has replaced all the parts of the frontal, temporal and parietal lobes and shifted the midline structures. Interictal EEG consist of generalized slow waves and dysrhythmia between two sides with left attenuation and transient sharp waves. Due to the stable mental status and no further motor function disturbance, the seizure was controlled by anti-epileptic drugs such as depakin 500 mg and levebel 500mg every 12 hours.

**Figure 1.A:**
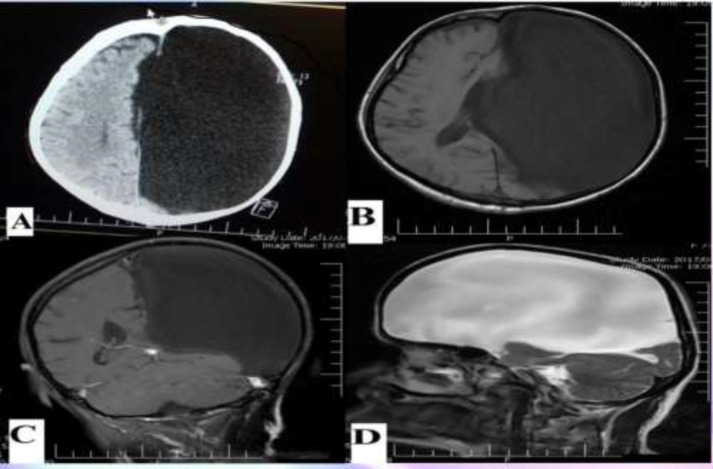
Axial non-contrast-CT scan showing a huge hypodense area in the left hemisphere of the brain

Figure 1.B: The same lesion in Tl weight MRI showing a huge unilocular cyst extending through left hemisphere

Figure 1.C: C. Coronal Tl -weighted MRI image with gadolinium. Figure 1.D: Sagittal T2-wieghted MRI.

## Discussion

Arachnoid cysts are not neoplastic masses and there is no association between arachnoid cysts and mortality (12). These cysts have congenital or acquired region, present no symptoms (2). The prevalence of arachnoid cysts in men is more than women and the common location is in middle fossa (12). Patients with arachnoid cyst maybe have headache, dizziness or seizure but there is no clear association between these symptoms and arachnoid cysts (8). The case reported by Mackle and Wile, referred to department with clinical symptoms of seizure. Radiographic imaging showed a large arachnoid cyst in right hemisphere which was chronic lesion. She was treated conservatively (with lamotrigine) and had no recurrent symptoms after 6 months (13). The definitive treatment for arachnoid cyst with progressive sign and symptoms and refractory epilepsy is surgery (14, 15). In an article by Birjandi et al., surgery was done on 20 cases with arachnoid cyst. Their most common signs and symptoms were refractory seizure, increased intracranial pressure, visual disorder. After 6 months of follow-up, the cyst size remained small and neither serious complication nor mortality in these patients is reported (16). These cysts are discovered incidentally in MRI or CT-scan (5). We aimed to report an unusual case who has the late onset epilepsia partialis continua in the 5^th^ decade of life with probably a large arachnoid cyst since newborn. Surprisingly without any history of seizure, her first seizure presented with sustained seizures (epilepsia partialis continua) and her seizures occur in the middle age for first time. She went under treatment after discovering arachnoid cyst in her MRI. She did not get surgery and her seizures were controlled completely with depakin 500 mg every 12 hours and levebel 500 mg every 12 hours, after a few days. She had no seizures after discharge of hospital and did not have new neurological deficit after that.

In conclusion most arachnoid cysts are asymptomatic and may not produce any symptoms throughout life. They are generally diagnosed incidentally on CT-scan and MRI. In our case, the late onset epilepsia partialis continua in the 5^th^ decade of life with probably a large arachnoid cyst without any history of seizure before that is unusual. Conservative approaches usually made for the management of arachnoid cysts as patients with these cysts usually maintain the vital neurological functions.

## References

[1] Bright R Report of medical cases, selected with a view of illustrating the symptoms and cure of diseases by a reference to morbid anatomy.

[2] Ariai S, Koerbel A, Bornemann A, Morgala M, Tatagiba M (2005). Cerebellopontine angle arachnoid cyst harbouring ectopic neuroglia. Pediatr Neurosurg.

[3] Gelabert-González M (2004). Intracranial arachnoid cysts. Rev Neurol.

[4] Gurkas E, Altan BY, Gücüyener K, Kolsal E (2015). Cerebellopontine angle arachnoid cyst associated with mirror movements. J Pediatr Neurosci.

[5] Yalçin AD, Oncel C, Kaymaz A, Kuloğlu N, Forta H (2002). Evidence against association between arachnoid cysts and epilepsy. Epilepsy Res.

[6] Westermaier T, Schweitzer T, Ernestus RI (2012). Arachnoid cysts. Adv Exp Med Biol.

[7] Clavel M, Taborga FG, Onzain I (1985). Arachnoid cysts as a cause of dementia in the elderly. Acta Neurochir (Wien).

[8] Rabiei K, Jaraj D, Marlow T (2016). Prevalence and symptoms of intracranial arachnoid cysts: a population-based study. J Neurol.

[9] Wester K, Wester K ( 2018 ). Intracranial arachnoid cysts and epilepsy. Arachnoid cysts.

[10] Pradilla G, Jallo G (2007). Arachnoid cysts: case series and review of the literature. Neurosurg Focus.

[11] Westermaier T, Schweitzer T, Ernestus RI (2012). Arachnoid cysts. In: Ahmad SI, editor. Neurodegenerative diseases. US, New York: Springer.

[12] Al-Holou WN, Terman S, Kilburg C (2013). Prevalence and natural history of arachnoid cysts in adults. J Neurosurg.

[13] Mackle T, Wile D (2017). Wile. Arachnoid cysts and adult onset epilepsy. CMAJ.

[14] Broekx S, Vandevenne J, Weyns FJM (2020). Exploring the controversial association between intracranial arachnoid cysts and epileptogenesis: a case report and review of the literature. SN Compr Clin Med.

[15] Gelabert-González M (2004). Intracranial arachnoid cysts. Rev Neurol.

[16] Birjandi AR, Etemadrezaee H, Samini F (2008). Intracranial arachnoid cyst (review of 20 cases). Iran J Otorhinolaryngol.

